# Epstein-Barr virus infection associated polymyositis and coronary artery dilation

**DOI:** 10.1186/s12879-022-07221-9

**Published:** 2022-03-07

**Authors:** Liping Teng, Chencong Shen, Weizhong Gu, Jianqiang Wu, Meiping Lu, Xuefeng Xu

**Affiliations:** 1 Department of Rheumatology Immunology & Allergy, The Children’s Hospital, Zhejiang University School of Medicine, National Clinical Research Center for Child Health, 310003 Hangzhou, People’s Republic of China; 2 Department of Pathology, The Children’s Hospital, Zhejiang University School of Medicine, National Clinical Research Center for Child Health, 310003 Hangzhou, People’s Republic of China; 3 Department of Pulmonary Medicine, The Children‘s Hospital, Zhejiang University School of Medicine, National Clinical Research Center for Child Health, 310003 Hangzhou, People’s Republic of China

**Keywords:** Children, Epstein-Barr virus, Polymyositis, Coronary artery dilation

## Abstract

**Background:**

Epstein-Barr virus (EBV) infects more than 90% of the population worldwide. However, chronic active EBV infection (CAEBV) is one of the EBV-positive T- or NK-lymphoproliferative diseases with high morbidity and mortality. Here, we report a case of a 9-year girl with CAEBV, successively presenting with polymyositis and coronary artery dilation (CAD).

**Case presentation:**

The girl complained of fatigue for more than 1 month. Muscle strength examinations had no abnormal findings. Blood chemistries showed elevated alanine aminotransferase (ALT), aspartate aminotransferase (AST), and creatine kinase (CK). Magnetic resonance imaging (MRI) showed spotty high-intensity signals in thigh muscles, and electromyogram suggested myogenic damage. The significant findings were positive EBV antibodies (EBVEA-IgG, EBVCA-IgG, and EBVNA-IgG), increased EBV DNA copies in B, T, and NK cells, and positive EBV-encoded small RNA in biopsy muscle specimen. The girl received ganciclovir, intravenous immunoglobulin, and methylprednisolone, and her symptoms improved. On the 45th day of hospitalization, echocardiograph revealed CAD. She received additional anticoagulants and Tocilizumab. Her condition improved and continued to be followed up at the clinic preparing for hematopoietic stem cell transplantation.

**Conclusions:**

This is the first reported case of CAEBV successively with polymyositis and CAD. This case makes the diagnoses of autoimmune diseases in children more complicated. Careful investigation of hidden CAEBV should be recommended in children with atypical polymyositis or CAD.

## Background

Epstein-Barr virus (EBV) is a ubiquitous double-stranded DNA virus, infecting more than 90% of the population worldwide [1]. Most of EBV infections induce subclinical or acute infectious mononucleosis in immunocompetent individuals with transient proliferation of EBV-infected B lymphocytes accompanied by excessive response of EBV-specific cytotoxic T lymphocytes [2]. However, chronic active EBV infection (CAEBV) is a severe disease with high morbidity and mortality, especially in East Asian countries. In CAEBV, EBV-infected T or natural killer (NK) cells clonally proliferate and infiltrate multiple organs, leading to fatal complications such as multi-organ failure, polymyositis, coronary artery aneurysms, vasculitis of the central nervous system, and malignant lymphomas as well as hemophagocytic lymphohistiocytosis [3–6].

No previous reports in the existing literature have definite cases of EBV infection manifesting as both polymyositis and coronary artery dilation (CAD). We described here a rare pediatric case with EBV infection, successively presenting with polymyositis and CAD. The case report aims to make pediatricians pay more attention to the complexity of pediatric cases with EBV infection.

## Case presentation

In July 2020, a 9-year girl was admitted to the Department of Gastroenterology in our hospital for fatigue for more than 1 month, and abnormal liver function for 2 days. Physical examination on admission revealed visible tonsils, and cervical lymphadenectasis; no rales in both lungs; no murmurs in her heart; no positive signs on nervous system; and no skin rash. The liver was palpated 3 cm below the costal margin. Muscle strength examinations had no abnormal findings. When she was 3 years old, she got thrombocytopenic purpura and received oral steroid therapy for 3 months.

**Fig. 1:**
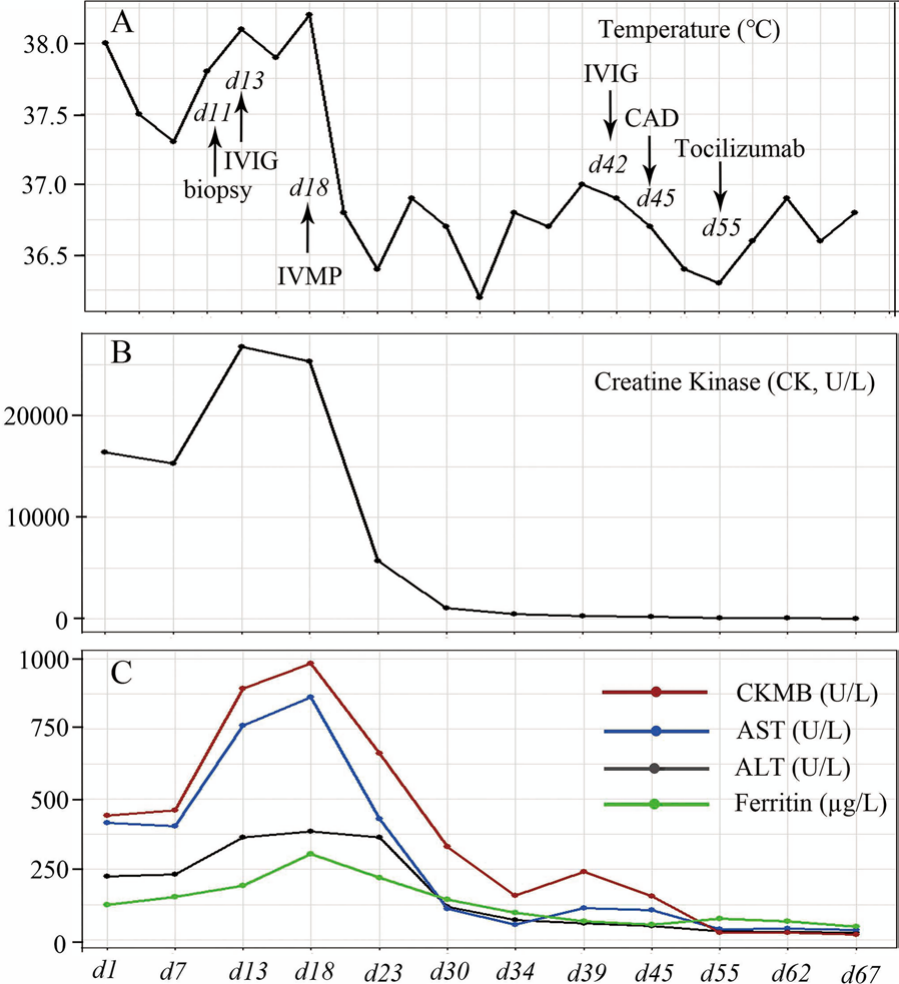
Clinical course of the patient. Patient’s body temperature (**A**), CK (**B**), CKMB, AST, ALT, and ferritin **C** significantly improved after IVIG and IVMP treatments. *ALT *alanine aminotransferase, *AST *aspartate aminotransferase, *CAD *coronary artery dilation, *CK *creatine kinase, *CKMB *creatine kinase MB, *IVIG *intravenous immunoglobulin, *IVMP *intravenous methylprednisolone

**Fig. 2:**
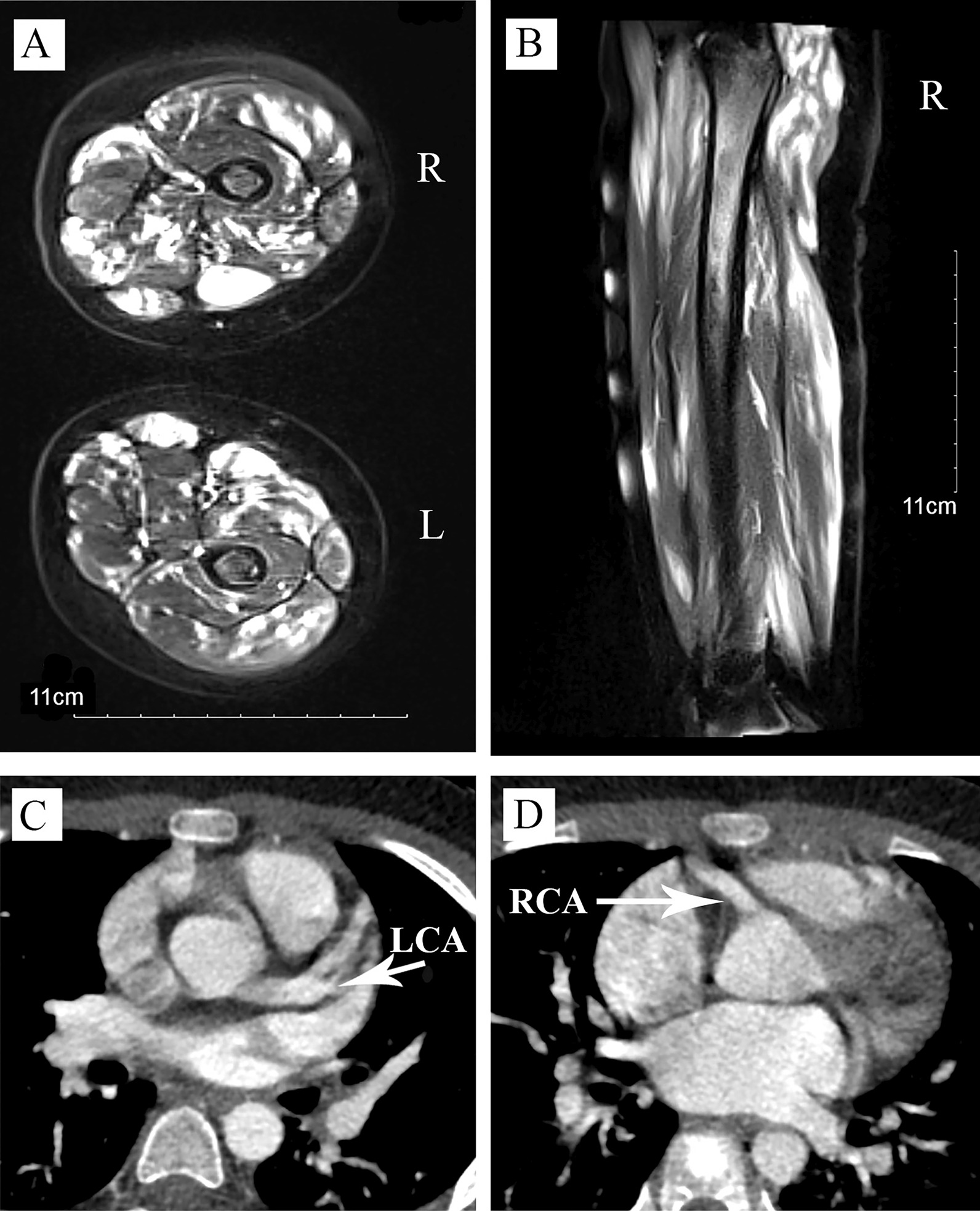
Imaging findings. The axial (**A**, right and left thighs) and sagittal (**B**, right thigh) T2-weighted magnetic resonance imaging (MRI) showed spotty high-intensity signal in thigh muscles such as adductor muscles, and femoral quadriceps muscles indicating muscle inflammation. Contrast-enhanced computed tomography angiography (CTA) showed left **C** and right **D** coronary artery dilations. *LCA *left coronary artery, *RCA *right coronary artery

Peripheral blood counts showed WBC 5.7 × 10^9^/L with 70% neutrophils, 23% lymphocytes, Hgb 102 g/L and a platelet count 223 × 10^9^/L. Blood chemistries indicated alanine aminotransferase (ALT) of 224 U/L, aspartate aminotransferase (AST) of 417 U/L, lactate dehydrogenase (LDH) of 1363 U/L, creatine kinase (CK) of 16,418 U/L, CKMB of 441 U/L, and ferritin of 123 mg/L (Fig. 1). EBV IgM and cytomegalovirus (CMV) IgM were negative, and EBVEA-IgG, EBVCA-IgG, EBVNA-IgG were positive (Table 1). Antinuclear antibodies, coagulation function, thyroid function, and immunoglobulin were within normal ranges. Hepatitis viruses B and C, and human immunodeficiency virus were negative. Detections of myositis specific antibodies were negative. Electromyogram suggested myogenic damage, and echocardiography showed no coronary artery dilation. Chest CT showed small patchy shadow in left upper lobe, and lung function testing was normal. The magnetic resonance imaging (MRI) showed spotty high-intensity signals in both of thigh muscles such as adductor muscles, and femoral quadriceps muscles indicating muscle inflammation (Fig. 2A). Bone marrow aspiration did not show leukemic changes and hemophagocytosis.

**Table 1:** Clinical characteristics of the patient on admission

Clinical variables	Values	References
IgG (g/L)	20.8	6.36–14.04
IgG1 (g/L)	24.8	4.9–11.4
IgG2 (g/L)	1.87	1.5–6.4
IgG3 (g/L)	0.99	0.2–1.1
IgG4 (g/L)	0.01	0.08–1.4
IgM (g/L)	2.49	0.29–1.21
IgA (g/L)	1.98	0.63–1.79
IgE (U/L)	4.3	< 100
CD 20 (%)	4.91	14–21
CD3 (%)	73.46	64–72.5
CD4 (%)	32.06	29.5–35.5
CD8 (%)	37.58	24–33.5
CD3–CD16 + CD56+ (%)	12.99	11–23
CD4/CD8	0.85	0.9–1.4
ANA	< 1:80	< 1:80
RF (U/ml)	255	0–14
EBVEA–IgG	+	–
EBVCA–IgG	> 750 U/mL	–
EBVCA–IgM	−	–
EBVNA–IgG	> 600 U/mL	–
CMV IgM	−	–
EBV DNA (copies/mL)	−	–
Plasma (day 7)	1.09 × 10^4^	< 5.0 × 10^2^
Plasma (day 23)	6.96 × 10^3^	< 5.0 × 10^2^
Plasma (day 39)	9.55 × 10^2^	< 5.0 × 10^2^
Plasma (day 55)	1.22 × 10^3^	< 5.0 × 10^2^
B cells (day 7, copies/10^6^ cells)	1.37 × 10^4^	< 5.0 × 10^2^
T cells (day 7, copies/10^6^ cells)	9.02 × 10^5^	< 5.0 × 10^2^
NK cells (day 7, copies/10^6^ cells)	1.64 × 10^4^	< 5.0 × 10^2^
Mitochondrial gene testing	No significant pathogenic gene mutations	–
Whole exome testing	No significant pathogenic gene mutations	–

*ANA* antinuclear antibody, *CMV* cytomegalovirus, *EBVEA* Epstain-Barr virus early antigen, *EBVCA* Epstain-Barr virus capsid antigen, *EBVNA* Epstain-Barr virus nuclear antigen. −, negative; +, positive

**Fig. 3:**
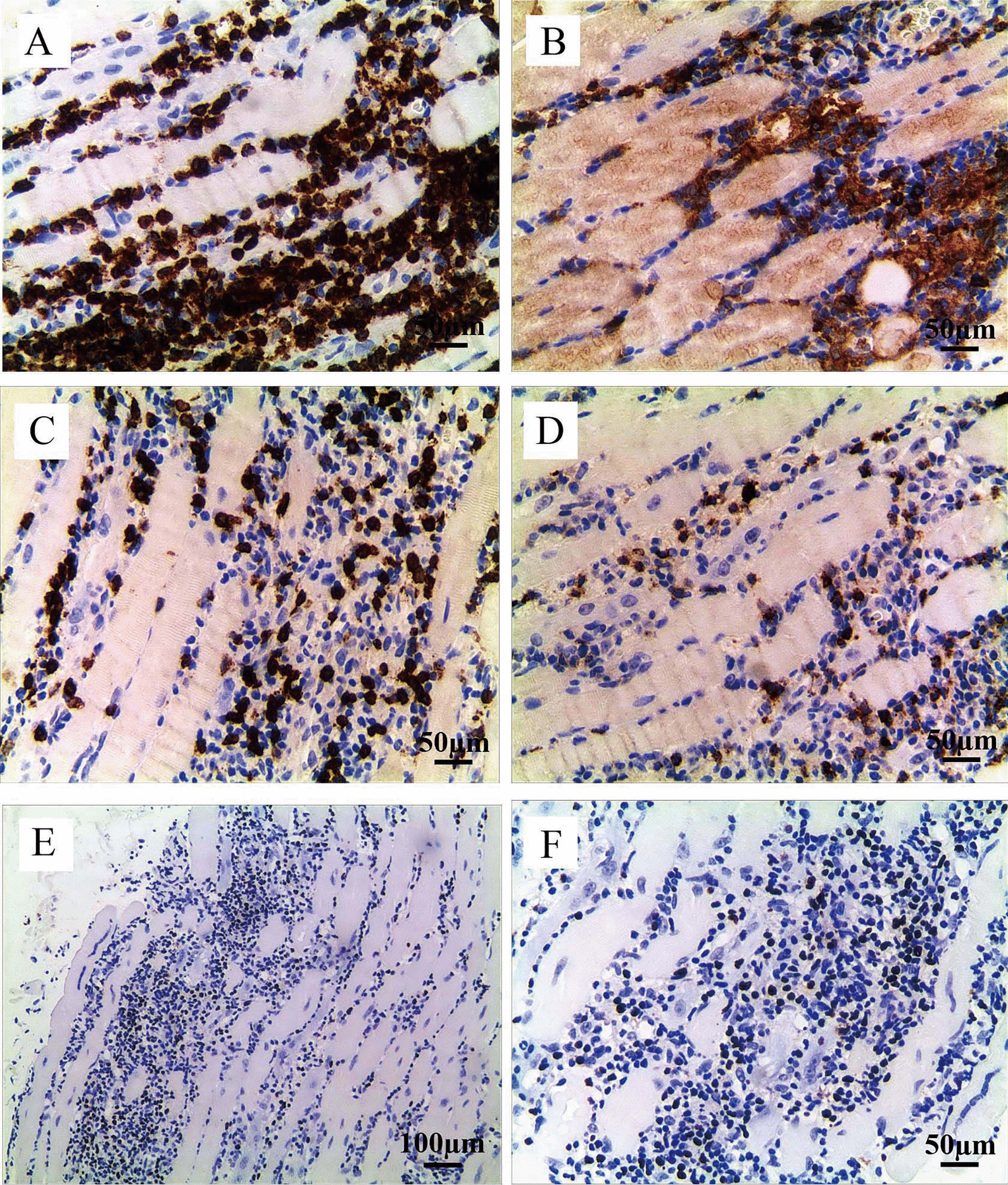
Histopathological findings of thigh muscle biopsy specimens. The inflammatory infiltrations were mainly composed of CD3+ (**A**, brown signal), CD4+ (**B**, brown signal), and CD8+ (**C**, brown signal) T lymphocytes, and CD20+ (**D**, brown signal) cells were also observed. The predominantly infiltrated lymphocytes were T cells. EBV-encoded small RNA (EBER) positivity was observed in the lymphocytes by in situ hybridization (**E**, **F**, brown signal)

One week after admission, the girl was suspected of polymyositis, and transferred to Department of Rheumatology and Immunology. Considering her inconsistence with typical polymyositis features, we further performed muscle biopsy and whole medical exons analysis. Histopathological findings of thigh muscle biopsy demonstrated a large amount of inflammatory cells infiltrations with predominant CD3+, CD4+, and CD8 + T lymphocytes (Fig. 3A–D). *In situ* hybridization indicated the expression of EBV-encoded small RNA (EBER) in the lymphocytes (Fig. 3E, F). EBV DNA detections in B, T, and NK cells were positive (Table 1). Whole body ^18^ F-FDG (Fluoro-D-Glucose) PET/MRI suggested multiple muscles’ patchy abnormal signals and an increased FDG metabolism. Although the girl had elevated EBV genome in peripheral B, T, and NK cells, positive EBER in biopsy muscle specimen, and abnormal liver function, her clinical symptoms only persisted for less than 2 months, which did not meet the diagnostic criteria of chronic active EBV infections (CAEBV). Therefore, she was diagnosed as EBV-related polymyositis, and treated with ganciclovir, intravenous immunoglobulin (once a month), methylprednisolone (1 mg/kg/d), and other supportive treatment. Twenty days after admission, her temperature turned normal, CK, CKMB, and ALT significantly reduced, and her condition improved (Fig. 1). However, on the 45th day of hospitalization, repeated echocardiograph revealed coronary artery dilation (CAD). The mean luminal diameters of left and right coronary arteries were 6.5 mm, and 5.8 mm, respectively. CAD was further confirmed by contrast-enhanced computed tomography angiography (CTA) without vascular lesions outside the heart (Fig. 2B, C). The girl was treated with oral aspirin and clopidogrel. During our consecutive echocardiograph monitoring, left and right coronary arteries were found to be progressively dilated (about 2 mm increases within a week). She was suspected of vasculitis and infused with Tocilizumab (8 mg/kg/month). Her CAD did not worsen, and continued anticoagulation and Tocilizumab treatments, while her oral steroids gradually tapered. After about 2 months of hospitalization, the patient’s symptoms improved, and her condition was stable. Therefore, she was discharged, and continued to be followed up at the clinic preparing for hematopoietic stem cell transplantation.

## Discussion and conclusions

Clinical diversification of EBV infections, especially CAEBV would make the diagnoses of autoimmune diseases more challenging. We herein described a pediatric case with EBV infection who successively presented with polymyositis and CAD. She was treated with steroid, regular immunoglobulin transfusion, and biologic agent.

The patient had high EBV DNA copies in peripheral B, T, and NK cells, positive EBER in muscle specimen, and abnormal liver function. Although her clinical symptoms on admission only persisted for less than 2 months, successive polymyositis and CAD suggested the presence of CAEBV. Skeletal muscle involvement in CAEBV is relatively rare, and polymyositis or generalized myositis is also rare [7]. In general, double negative (DN, CD4- and CD8-) T-cells do not populate in normal human muscles. However, EBV + DN T-cells infiltrated muscle tissues during the course of CAEBV, resulting in myositis [3]. Some patients showed steroid non-responsive chronic progressive generalized myositis, predominantly intramuscular infiltrating CD3+, EBER+, DN T cells [7]. This pattern is significantly different from CD8+ (single positive) T cell dominance in polymyositis, and CD4 + T cell dominance in dermatomyositis [7]. Kobayashi et al. reported a case of CAEBV with EBER+, CD8 + T cell infiltrations in the brachialis muscle specimen. The patient was treated with prednisone and immunosuppressant, and her CK levels and EBV-DNA copies of the peripheral mononuclear cells rapidly decreased [8]. There are few reports about CAEBV combined with myositis. Our case showed predominant CD3+, CD4+, and CD8 + T lymphocytes infiltrations in muscle specimen with positive EBER, further confirming CAEBV. Her elevated CK and symptoms significantly improved through steroid and antiviral treatments. Unfortunately, as the polymyositis improved, she developed coronary artery dilation.

About 10% pediatric cases with CAEBV had cardiovascular complications, including pericarditis, myocarditis, and mitral valve regurgitation [9]. The cardiovascular lesions also presented with pulmonary arterial hypertension and systemic vasculitis [10]. Fujimoto *et at.* reported two cases of ulcerative vasculitis in methotrexate-prescribed rheumatoid arthritis patients, simulating rheumatoid vasculitis [11]. EBER on their skin biopsies confirmed the diagnosis of EBV-related vasculitis instead of rheumatoid vasculitis [11]. Additionally, a high positive rate of antineutrophil cytoplasmic autoantibody (ANCA) in the sera positive for EBV IgM suggested that EBV infection might trigger the relapse of ANCA-associated vasculitis [12]. Furthermore, the brain biopsy from a 75-year woman with EBV-associated vasculitis who had a history of rheumatoid arthritis, showed perivascular lymphocytic infiltration with EBER in the parenchyma and meninx [6]. This further indicated that EBV can almost invade all the vessels in the body.

Notably, CAEBV may cause aneurysmal dilation of the lumens in other lager vessel such as carotid and subclavian arteries, abdominal aorta and its major branches, in addition to coronary arteries [13]. In view of the EBER-positive lymphocytes detected in the vasa vasorum, these arterial lesions were obviously different from Kawasaki disease [13]. Myocardial EBV infection can also induce acute myocarditis with heart failure, necrotizing coronary vasculitis, and multiple left ventricular aneurysms. The elevated myocardial viral load, positive EBV protein in cardiomyocytes, and inflamed coronary intramural vessels confirmed the causal role of EBV [14]. Cardiovascular lesions in CAEBV could be strongly associated with EBV-infected T or NK lymphocyte infiltration and injuries in the vessel walls, and EBV-induced high levels of inflammatory cytokines [5]. Our case developed CAD when her clinical symptoms improved. Furthermore, ten days after confirming CAD, plasma EBV DNA copies slightly increased from 9.55 × 10^2^ to 1.22 × 10^3^. This phenomenon could indicate the direct infiltrations of EBV-infected lymphocytes into vascular wall or immune dysfunction induced by EBV. Since we did not perform histological examination of the damaged coronary arteries, the underlying mechanism needs to be further investigated. On the other hand, our patient only presented with mild fever and swollen lymph nodes in the neck, which did not meet the diagnosis of Kawasaki disease. Thus, it is reasonable to speculate that CAD might be caused by vasculitis induced by EBV infection instead of Kawasaki disease.

## Conclusions

Patients with CAEBV may have various symptoms and clinical courses. We herein reported the first case of CAEBV successively presenting with polymyositis and CAD. This case further suggests that CAEBV could make the diagnoses of autoimmune diseases in children more complicated. Careful investigation of hidden CAEBV should be recommended in children with atypical polymyositis or coronary artery dilation.

## Data Availability

The raw data supporting the conclusions of this article will be made available by the authors, without undue reservation.

## Abbreviations

CAEBV: Chronic active Epstein-Barr virus infection
CAD: Coronary artery dilation
CD: Cluster of differentiation
EBER: EBV-encoded small RNA
EBV: Epstein-Barr virus
NK: Natural killer
